# Platelet-derived growth factor (PDGF) and its impact in fibroid pathogenesis: A narrative review

**DOI:** 10.1097/MD.0000000000042995

**Published:** 2025-06-20

**Authors:** Emmanuel Ifeanyi Obeagu

**Affiliations:** a Department of Biomedical Laboratory Science, Africa University, Mutare, Zimbabwe.

**Keywords:** fibroid pathogenesis, growth factors, PDGF signaling pathways, platelet-derived growth factor, therapeutic targets, uterine leiomyomas

## Abstract

Uterine fibroids, also known as leiomyomas, are benign tumors of the uterus that disproportionately affect women of reproductive age, often leading to significant clinical symptoms such as abnormal bleeding, pelvic pain, and infertility. Although hormones like estrogen and progesterone have long been established as major contributors to fibroid development, recent advances have shed light on the critical role of growth factors in modulating tumor growth and fibrosis. Among these, platelet-derived growth factor (PDGF) has emerged as a key mediator of cellular proliferation, extracellular matrix deposition, and angiogenesis within fibroid tissues. PDGF exists in several isoforms (AA, BB, AB, CC, DD) and signals through PDGF receptors α and β, activating downstream pathways such as PI3K/AKT, RAS/MAPK, and JAK/STAT. These signaling events promote not only smooth muscle cell proliferation but also fibrotic remodeling by stimulating collagen and fibronectin production. PDGF also interacts with other pathways (particularly transforming growth factor-beta and sex hormones) to amplify fibroid growth and resistance to apoptosis, reinforcing a fibrotic, hormone-responsive microenvironment. Elevated expression of PDGF and its receptors in fibroids compared to normal myometrium supports its central role in pathogenesis.

## 1. Introduction

Uterine fibroids, or leiomyomas, are the most common benign tumors affecting women of reproductive age, with a prevalence of up to 70% by the age of 50. These tumors arise from the smooth muscle cells of the myometrium and are characterized by a well-circumscribed, fibrous mass that may vary in size and number. While many fibroids are asymptomatic, a significant portion of affected women experience symptoms such as heavy menstrual bleeding, pelvic pressure or pain, frequent urination, and reproductive dysfunction, including infertility and pregnancy complications.^[1,2]^ The pathogenesis of uterine fibroids is multifactorial, involving an interplay of genetic predisposition, hormonal influences, and environmental exposures. Estrogen and progesterone are key hormonal drivers of fibroid growth, stimulating both smooth muscle cell proliferation and extracellular matrix (ECM) accumulation. However, hormonal regulation alone does not fully explain the complexity of fibroid development. Increasingly, research has turned attention to the role of growth factors and signaling molecules that modulate the fibrotic and proliferative behavior of fibroid cells.^[3,4]^ Among the diverse array of growth factors implicated in fibroid pathophysiology, platelet-derived growth factor (PDGF) has emerged as a particularly potent mitogen and profibrotic agent. PDGF is a multifunctional peptide primarily known for its role in wound healing, angiogenesis, and mesenchymal cell proliferation. Dysregulation of PDGF signaling has been implicated in various fibrotic diseases and tumors, making it a focal point of interest in uterine fibroid research. Its expression is markedly upregulated in fibroid tissues compared to normal myometrium, suggesting a pivotal role in the disease process.^[5,6]^ PDGF is synthesized as a family of disulfide-linked dimers composed of A, B, C, and D chains, forming isoforms such as PDGF-AA, PDGF-BB, PDGF-AB, PDGF-CC, and PDGF-DD. These isoforms exert their biological activity by binding to and activating 2 tyrosine kinase receptors: PDGF receptor-alpha and PDGF receptor-beta. Ligand–receptor interaction induces receptor dimerization and autophosphorylation, initiating a cascade of downstream signaling pathways, including PI3K/AKT, RAS/MAPK, and JAK/STAT. These pathways are critically involved in regulating cell proliferation, migration, survival, and ECM production.^[7,8]^

In uterine fibroids, PDGF acts as a key regulator of abnormal smooth muscle cell growth and fibrogenesis. It stimulates fibroblast and myofibroblast activity, promotes the deposition of collagen and fibronectin, and supports the formation of a dense ECM that characterizes fibroid tissue. This fibrotic matrix not only contributes to the physical bulk of the tumor but also modifies cellular behavior and mechanical signaling within the fibroid microenvironment. As a result, PDGF contributes to both tumor growth and the symptoms associated with fibroid pathology.^[9]^ Additionally, PDGF signaling is modulated by and interacts with other cellular pathways and factors that further amplify its effects. Notably, estrogen and progesterone have been shown to upregulate platelet-derived growth factor receptor (PDGFR) expression in fibroid cells, thereby sensitizing them to PDGF stimulation. PDGF also synergizes with transforming growth factor-beta, another key fibrogenic cytokine, to promote tissue remodeling and fibrosis. This crosstalk between hormonal and growth factor signaling pathways underscores the complex regulatory network driving fibroid pathogenesis.^[10]^ Current treatment options for uterine fibroids include pharmacologic therapies such as hormonal agents, minimally invasive procedures like uterine artery embolization, and surgical interventions including myomectomy and hysterectomy. However, these approaches are often associated with limitations, such as temporary symptom relief, impact on fertility, or procedural risks. Therefore, there is a growing need for novel, targeted therapies that address the underlying molecular mechanisms of fibroid development (PDGF being a promising target in this regard).^[11]^

### 1.1. Aim

The aim of this review is to provide an in-depth understanding of the role of PDGF in the pathogenesis of uterine fibroids.

## 2. Review methods

This review article was developed through a systematic synthesis of existing literature on PDGF and its role in the pathogenesis of uterine fibroids. The methodology employed in this review consisted of comprehensive database searches, critical selection of relevant articles, and synthesis of the findings to provide an updated understanding of PDGF’s role in fibroid biology.

### 2.1. Literature search strategy

A comprehensive literature search was conducted using multiple scientific databases, including PubMed, Google Scholar, Scopus, and Web of Science. The search terms included combinations of “Platelet-Derived Growth Factor,” “fibroid pathogenesis,” “leiomyoma,” “growth factor signaling,” “fibrosis,” “extracellular matrix,” and “uterine tumors.” The search was not limited by language, but articles were selected based on their relevance, publication date (primarily from the last 15 years), and scientific rigor. Articles covering PDGF’s molecular mechanisms, receptor interactions, signaling pathways, and implications in fibroid progression were prioritized.

### 2.2. Inclusion and exclusion criteria

The inclusion criteria for this review included peer-reviewed articles, clinical trials, and experimental studies that examined the role of PDGF in fibroid pathogenesis, particularly focusing on its signaling pathways, cellular effects, and potential therapeutic implications. Articles that specifically evaluated PDGF expression in uterine fibroids, interactions with other growth factors or hormones, and the molecular signaling pathways involved were included. Studies that investigated PDGF-targeted therapies or agents in fibroid treatment were also incorporated. Exclusion criteria involved the removal of articles that did not directly address PDGF in the context of uterine fibroids, studies with methodological flaws, or those that did not contribute significant new findings to the topic. Articles focused solely on other benign uterine conditions or those with limited relevance to PDGF in fibroid pathogenesis were excluded from the review.

### 2.3. Ethical approval

No ethical approval was obtained as this is a narrative review

## 3. Platelet-derived growth factor

PDGF is a family of proteins that play a crucial role in various cellular processes, including cell growth, proliferation, and differentiation. These growth factors are named for their initial discovery in platelets, but they are also produced by other cell types, such as endothelial cells, smooth muscle cells, fibroblasts, and macrophages.^[12]^ The PDGF family comprises several isoforms, including PDGF-AA, PDGF-BB, PDGF-AB, PDGF-CC, and PDGF-DD. Each isoform is a dimeric protein composed of 2 chains (A or B) linked together. These isoforms exert their effects by binding to specific tyrosine kinase receptors, primarily PDGFR-alpha (PDGFR-α) and PDGFR-beta (PDGFR-β), present on the cell surface.^[11]^ PDGF promotes the growth and division of various cell types, such as fibroblasts, smooth muscle cells, and vascular endothelial cells. This function is crucial for tissue repair, wound healing, and normal development. PDGF influences the production of ECM components like collagen and fibronectin. It plays a role in tissue remodeling, including wound healing and tissue repair processes. PDGF is involved in stimulating the formation of new blood vessels (angiogenesis). It acts as a chemoattractant for endothelial cells and supports vessel growth and stability. PDGF regulates cell migration and differentiation, influencing the behavior of various cell types during development, tissue repair, and pathological conditions.^[11]^ The dysregulation of PDGF signaling has been implicated in various diseases, including cancers, fibrotic disorders, and vascular diseases. In the context of fibroid pathogenesis, PDGF has been recognized for its involvement in promoting cellular proliferation, modulating ECM components, and contributing to the growth and maintenance of uterine leiomyomas.^[13]^ Targeting PDGF signaling pathways has been explored as a potential therapeutic strategy in different disease conditions. In the context of fibroids, understanding the specific actions of PDGF isoforms and their interactions with PDGF receptors may offer insights into novel approaches for managing and treating these benign tumors, potentially providing avenues for targeted interventions and personalized therapies.^[14]^

### 3.1. Fibroid

Fibroids, medically termed uterine leiomyomas, are noncancerous growths that develop in the muscular wall of the uterus. These growths are common among women of reproductive age and are typically benign, although they can cause various symptoms and complications.^[2]^ Fibroids are among the most common gynecological conditions, affecting a significant number of women. They can vary in size, number, and location within the uterine wall.^[3]^ Not all fibroids cause symptoms, but when they do, common symptoms may include heavy menstrual bleeding (menorrhagia), pelvic pain or pressure, frequent urination, prolonged menstrual periods, backache, and in some cases, reproductive issues such as infertility or recurrent miscarriages. Fibroids are classified based on their location within the uterus: subserosal fibroids grow on the outer wall, intramural fibroids develop within the muscular wall, and submucosal fibroids grow into the uterine cavity. The exact cause of fibroids remains unclear, but hormonal factors, particularly estrogen and progesterone, play a significant role in their growth. Genetic predisposition, hormonal imbalances, and other factors like race, family history, and obesity may also contribute to their development.^[5]^

Diagnosis often involves a pelvic exam, imaging studies such as ultrasounds, MRI, or hysteroscopy, and sometimes, biopsies to confirm the presence of fibroids and rule out other conditions.^[4]^ Treatment options vary based on the size, number, and location of fibroids, as well as the severity of symptoms and a woman’s reproductive plans. Treatment may include medication to manage symptoms, hormonal therapy, minimally invasive procedures like uterine artery embolization or focused ultrasound surgery, and in severe cases, surgical removal of the fibroids (myomectomy) or the entire uterus (hysterectomy).^[6]^ While fibroids are generally benign and nonlife-threatening, they can significantly impact a woman’s quality of life, causing discomfort, pain, and reproductive health issues. Ongoing research aims to better understand the underlying mechanisms of fibroid development, improve diagnostic techniques, and develop more targeted and less invasive treatment options for affected individuals.

### 3.2. Overview of fibroid pathogenesis

The pathogenesis of fibroids, also known as uterine leiomyomas, is a multifaceted process influenced by various factors, including hormonal, genetic, and environmental elements.^[15]^ Hormonal factors, particularly estrogen and progesterone, play a central role in the growth and development of fibroids. Estrogen, in particular, stimulates the proliferation of uterine smooth muscle cells, which constitute the bulk of fibroids. Progesterone also contributes to fibroid growth by promoting cell division and ECM production.^[16]^ There is a genetic component to fibroid development, as the risk of developing fibroids appears to run in families. Several genes have been identified as potentially associated with a higher risk of fibroids, although the precise genetic mechanisms contributing to their formation remain an area of ongoing research.^[17]^ Fibroids result from the abnormal growth of smooth muscle cells in the uterine wall. Changes in the ECM components, including collagen and other proteins, contribute to the formation and growth of fibroids. Dysregulation in signaling pathways involving growth factors, cytokines, and cellular receptors also influences fibroid pathogenesis.^[2]^ While less understood, environmental factors such as diet, obesity, and exposure to certain chemicals may influence fibroid development. Obesity, for instance, is associated with increased estrogen levels, potentially contributing to the growth of fibroids.^[4]^ Changes in the microenvironment of the uterus, including alterations in blood supply, inflammation, and oxidative stress, can also influence the initiation and growth of fibroids. The interplay between these factors contributes to the initiation and growth of fibroids. Hormonal imbalances, genetic predispositions, and environmental factors can disrupt the delicate balance in the uterus, leading to the uncontrolled proliferation of smooth muscle cells and the formation of fibroids.^[18]^ Advancements in research continue to elucidate the complex mechanisms involved in fibroid pathogenesis.

## 4. PDGF signaling pathways in fibroid development

The PDGF signaling pathways play a significant role in fibroid development, influencing various cellular processes crucial to the growth and maintenance of uterine leiomyomas. PDGF, existing as different isoforms, interacts with specific receptors, leading to downstream signaling cascades that contribute to the pathogenesis of fibroids.^[11]^ PDGF isoforms (PDGF-AA, PDGF-BB, PDGF-AB, PDGF-CC, and PDGF-DD) interact with 2 main receptors: PDGFR-α and PDGFR-β. These isoforms have varying affinities for these receptors, resulting in diverse cellular responses.^[19]^ PDGF signaling promotes the proliferation of uterine smooth muscle cells, a primary component of fibroids. Binding of PDGF isoforms to their receptors activates downstream signaling pathways that stimulate cell division and growth, contributing to the enlargement of fibroid tumors.^[20]^ PDGF influences the production and breakdown of the ECM within fibroids. It regulates the expression of enzymes such as matrix metalloproteinases and tissue inhibitors of metalloproteinases, affecting the turnover of ECM components like collagen and fibronectin.^[13]^ PDGF signaling contributes to the formation of new blood vessels (angiogenesis) within fibroid tissues. It acts as a chemoattractant for endothelial cells and plays a role in vessel growth and stabilization, ensuring adequate blood supply to the growing fibroid mass.^[20]^ PDGF signaling pathways influence cell migration and differentiation, impacting the behavior of various cell types within the fibroid microenvironment. This may contribute to the maintenance and progression of fibroids.^[13]^ PDGF interacts with other growth factors, cytokines, and signaling molecules within the fibroid milieu, leading to complex crosstalk between different pathways. These interactions further influence cellular responses and contribute to the overall environment supporting fibroid growth.^[11]^ The dysregulation of PDGF signaling pathways can result in aberrant cell proliferation, altered ECM dynamics, increased angiogenesis, and other cellular changes associated with fibroid development. Targeting PDGF receptors or downstream signaling molecules has been explored as a potential therapeutic approach to inhibit fibroid growth or mitigate symptoms associated with these tumors.^[19]^

## 5. Therapeutic implications and future directions

The recognition of the PDGF signaling pivotal role in fibroid pathogenesis holds significant therapeutic implications and suggests several future directions for managing uterine leiomyomas.^[21]^ Exploring targeted therapies aimed at disrupting PDGF signaling pathways offers promise in impeding fibroid growth. Inhibitors targeting PDGF receptors or downstream effectors could be investigated for their efficacy in limiting cellular proliferation and ECM remodeling within fibroids.^[22]^ Investigating combination therapies that target multiple pathways involved in fibroid development, including PDGF signaling, could potentially yield synergistic effects, enhancing treatment outcomes while minimizing adverse effects.^[23]^ Advancing research into personalized treatment approaches based on an individual’s unique PDGF receptor expression profile or genetic predisposition may lead to more tailored and effective therapeutic strategies. Encouraging the development of novel drugs or therapeutics specifically targeting PDGF pathways in fibroids warrants attention. Conducting well-designed clinical trials to evaluate the safety and efficacy of these targeted therapies in human subjects is essential.^[24]^ Identifying reliable biomarkers associated with PDGF activity or specific PDGF isoform expression within fibroids could aid in predicting treatment responsiveness and disease progression. This may facilitate early interventions and personalized treatment decisions.^[25]^

Investigating immunotherapeutic strategies, including targeted immunomodulation aimed at PDGF-related pathways, could present innovative avenues for managing fibroids. Emphasizing the importance of long-term monitoring and follow-up studies in clinical trials to assess the durability, recurrence rates, and long-term effects of PDGF-targeted therapies is crucial. Promoting collaboration among diverse scientific disciplines, sharing research findings, and pooling resources internationally can accelerate understanding PDGF-related mechanisms in fibroid pathogenesis and their clinical applications. Advocating for increased awareness about fibroids, their impact on women’s health, and the potential for novel therapies targeting PDGF pathways can lead to better support for research funding, improved patient care, and policy changes.^[26]^ The therapeutic implications of PDGF signaling in fibroid pathogenesis offer a promising landscape for innovative treatments. The translation of these insights into clinical applications requires collaborative efforts, rigorous research endeavors, and a multifaceted approach toward personalized and targeted interventions for individuals affected by uterine fibroids (Table 1).

**Table 1:** Some of the current drugs available for the treatment of fibroid pathogenesis.

Drug name	Class/mechanism of action	Indication	Mode of action	Limitations/side effects
Levonorgestrel IUD	Progestin (hormonal therapy)	Heavy menstrual bleeding associated with fibroids	Suppresses endometrial growth, reduces bleeding, shrinks fibroids due to progestin effects on the uterus	Irregular bleeding, uterine perforation, hormonal side effects (headache, mood changes, acne)
GnRH agonists (e.g., leuprolide, Goserelin)	Gonadotropin-releasing hormone (GnRH) agonists	Fibroid shrinkage, symptomatic relief	Decreases estrogen and progesterone levels, leading to temporary fibroid shrinkage	Menopausal symptoms (hot flashes, bone loss), loss of libido, vaginal dryness, osteoporosis risk
Ulipristal acetate	Selective progesterone receptor modulator (SPRM)	Symptom management of fibroids, presurgical shrinkage	Inhibits progesterone receptors, reducing fibroid size and bleeding	Liver toxicity risk, irregular bleeding, headache, nausea, and fatigue
Mifepristone	Antiprogestin (progesterone receptor antagonist)	Symptom management, presurgical shrinkage	Blocks progesterone receptors, reducing fibroid size and bleeding	Heavy bleeding, abdominal pain, fatigue, and potential adverse effects on the endometrium
Tranexamic acid	Antifibrinolytic	Heavy menstrual bleeding due to fibroids	Inhibits fibrinolysis, reducing menstrual blood loss without affecting fibroid size	Gastrointestinal upset, headache, potential for thromboembolic events if used long-term
Myomectomy (surgical option)	Surgical removal of fibroids	Symptomatic fibroids affecting fertility or causing significant symptoms	Surgical excision of fibroids, restores uterine anatomy and function	Invasive procedure, risk of infection, scar tissue formation, potential impact on fertility, recurrence of fibroids
Uterine artery embolization (UAE)	Interventional radiology procedure	Symptomatic fibroids (e.g., heavy bleeding)	Blocks blood supply to fibroids, leading to ischemic degeneration and shrinkage	Pain, infection, premature menopause risk, fibroid recurrence after treatment
Fibroid-specific monoclonal antibodies (in development)	PDGF, TGF-β, VEGF inhibitors (experimental)	Potential future treatments for fibroids	Target specific growth factors and signaling pathways involved in fibroid development	Limited data on long-term safety and efficacy, currently experimental and undergoing clinical trials

*Hormonal treatments* (such as GnRH agonists, levonorgestrel IUD, mifepristone, and ulipristal acetate) are commonly used to reduce fibroid size and alleviate symptoms like heavy bleeding but are typically only effective while the patient is using the medication. *Nonhormonal treatments*, like *tranexamic acid*, help manage symptoms but do not address the underlying cause of fibroid growth. *Interventional methods* like *myomectomy* and *uterine artery embolization* (*UAE*) are useful for patients seeking more permanent solutions, especially those who wish to preserve fertility.

## 6. Implication for clinical and health policy making

The implications of understanding the role of PDGF signaling in fibroid pathogenesis extend beyond clinical practice to encompass health policy-making considerations:

*Improved clinical management strategies*: Knowledge of PDGF’s involvement in fibroid development facilitates the development of more targeted and effective clinical management strategies. This includes personalized treatment approaches and the potential for tailored therapies based on individual PDGF expression profiles or genetic predispositions.

*Enhanced treatment outcomes*: Utilizing PDGF-targeted therapies or interventions can potentially improve treatment outcomes for individuals affected by fibroids. These targeted approaches may lead to better symptom management, reduced tumor growth, and minimized side effects compared to traditional treatments.

*Precision medicine integration*: Integrating the understanding of PDGF-related pathways into the broader framework of precision medicine allows for tailored treatments specific to each patient’s molecular and genetic profile. This integration can revolutionize fibroid management and optimize patient outcomes.

*Clinical trial design and funding allocation*: Encouraging the design of clinical trials focused on PDGF-targeted therapies and supporting research funding directed toward investigating PDGF-related interventions can pave the way for innovative treatments and validate their efficacy in clinical settings.

*Healthcare resource allocation*: Health policy-makers need to consider the implications of adopting novel PDGF-targeted therapies in healthcare systems. Assessing the cost-effectiveness and resource implications of these treatments is vital for their integration into routine clinical practice.

*Patient access and affordability*: Health policies should address patient access to PDGF-targeted therapies, ensuring affordability, equitable access, and coverage under healthcare insurance plans to provide these advanced treatments to those in need.

*Regulatory framework and approval processes*: Health policies should adapt regulatory frameworks to accommodate the approval and safe implementation of PDGF-targeted therapies. This includes streamlining approval processes and ensuring stringent safety evaluations.

*Education and awareness initiatives*: Health policies should support educational initiatives to raise awareness among healthcare providers, patients, and the general public about fibroids and the potential benefits of PDGF-targeted therapies. This could lead to earlier diagnosis, better-informed treatment decisions, and improved patient outcomes.

*Data sharing and collaboration*: Policy initiatives that encourage data sharing, collaboration among researchers, and international partnerships can expedite the translation of research findings into clinical applications and foster a global effort to combat fibroids effectively.

## 7. Conclusion

PDGF plays a pivotal role in the pathogenesis of uterine fibroids through its involvement in cell proliferation, ECM deposition, and fibrotic remodeling. As a potent mitogen and fibrogenic agent, PDGF influences smooth muscle cell behavior and promotes the formation of a fibrotic microenvironment within fibroids. The dysregulated expression of PDGF and its receptors in fibroid tissues underscores its central role in tumor growth and the persistence of fibroid symptoms. The complex interplay between PDGF signaling, hormonal regulation by estrogen and progesterone, and interactions with other growth factors such as transforming growth factor-beta further complicates the pathophysiology of uterine fibroids. Understanding these molecular mechanisms provides valuable insights into how fibroid growth is regulated at a cellular level and how PDGF could be targeted for therapeutic interventions.

## Author contributions

**Conceptualization:** Emmanuel Ifeanyi Obeagu.

**Methodology:** Emmanuel Ifeanyi Obeagu.

**Supervision:** Emmanuel Ifeanyi Obeagu.

**Visualization:** Emmanuel Ifeanyi Obeagu.

**Writing – original draft:** Emmanuel Ifeanyi Obeagu.

**Writing – review & editing:** Emmanuel Ifeanyi Obeagu.
